# Inclusive service learning: contributions from an acrosport programme for university students with and without intellectual disabilities

**DOI:** 10.3389/fpsyg.2025.1648931

**Published:** 2025-11-19

**Authors:** Raquel Aguado-Gómez, María-Teresa Calle-Molina, Ismael Sanz-Arribas

**Affiliations:** Department of Physical Education, Sport, and Human Movement, Autonomous University of Madrid, Madrid, Spain

**Keywords:** cooperative sport, adapted physical activity, socialisation, teacher education, higher education, disability

## Abstract

In an increasingly globalised and diverse world, inclusion is a crucial pillar of social development. Creating environments where everyone feels valued, respected, and supported is important for personal and social development. This article analyses the possibilities for training and inclusion provided by an acrosport service learning project based around shared learning by all participants. University students from a physical activity and sports sciences (PASS) programme participated in this project, implementing an inclusive acrosport training programme for students with intellectual disabilities from the university’s own Training for Labour Market Inclusion (TLMIC) and for students from the bachelor’s degree in primary education with a specialisation in physical education (BPE-PE). This research used a qualitative methodological design that made it possible to approach the participants’ perspective. A variety of information collection instruments were used: individual interviews, a focus group, field journals, and learning journals. The results showed that participating in inclusive environments, which promote the active, equitable, and meaningful participation of all students within a shared learning environment, benefited the participating students with intellectual disabilities and the future PE teachers. The former group learned about acrosport and positively valued the sessions’ inclusiveness as this enabled them to meet new people and make connections outside class in a university setting with people who do not have disabilities, and they experienced feelings that they identified as positive. As for the students without disabilities who played the role of teachers, they felt that their learning of the content was more meaningful and they felt more self-confident when designing and implementing inclusive sessions. Finally, the participants without disabilities (in the roles of teacher and student) felt that they had transference at an academic and professional level of what they learned and that the inclusive experience made them reject stereotypes about people with disabilities. They also regarded having established links with people from various programmes as being positive and experienced positive feelings from having participated in the project. The findings of this research suggest that the development of the inclusive acrosport service learning programme generates relevant benefits for all those involved.

## 1 Introduction

In recent years and in different countries, there has been growth in post-compulsory training programmes delivered in the university setting (Berastegui et al., 2023). These programmes are centred around developing learning and skills that facilitate the vocational integration of all of the population, including people with disabilities. The development of these programmes focusses on learning concepts and procedural skills and, above all, on developing self-determination and self-efficacy, acquiring social skills and the effective inclusion of all people in society (Berastegui et al., 2015; Izuzquiza, 2011; Izuzquiza-Gasset and Rodríguez-Herrero, 2016; Marttinen et al., 2020).

Specifically, there are university programmes for people with intellectual disabilities. Their purpose is to educate people with intellectual disabilities to acquire competencies and skills that will help them later enter the workforce. In addition, inclusion in a university environment makes people with intellectual disabilities visible amongst the university student body. Inclusion reduces the prejudices and cultural barriers that can exist in the university community regarding people with disabilities because socialisation, normalised contact under equal conditions, is fostered between people who do and do not have disabilities inside and outside class (Berastegui et al., 2015; Klavina et al., 2014). This type of initiative also improves empathy, increases positive attitudes, reduces discrimination and increases the capacity to resolve the difficulties and barriers that students with disabilities face in a university setting, and it reduces prejudices towards this group (Lumish et al., 2022; Park et al., 2024). In short, community participation and learning opportunities for people with intellectual disabilities are fostered (Lawson et al., 2017; Rickson and Warren, 2018; Sánchez-Díaz and Morgado, 2022; Toutain, 2019).

University service learning (USL) is a useful tool for promoting the inclusion of different groups of people in the academic sphere because it enables people with different realities and backgrounds to relate in a natural and meaningful way (Case et al., 2020). Research has shown that these projects inspire positive learning and changes in attitude amongst university students and teachers as they help to increase their professional competences, their empathy, their comprehension, and their awareness of people with disabilities and of the challenges that society poses for them (Bringle and Clayton, 2021; García-Rico et al., 2023; Lawson et al., 2017; Woodruff and Sinelnikov, 2015). Furthermore, USL projects improve university students’ ability to understand and adapt to the characteristics and needs of service users. Consequently, exposure to these contexts of real practise reduces university students’ anxiety and uncertainty when meeting the people who use the service, who often live very different lives from those found in the university context (Santiago et al., 2020; Marttinen et al., 2020; Lynch et al., 2019; Pettigrew and Tropp, 2008).

There is abundant scientific evidence that SL has a positive impact on university students’ learning. The promotion of intentional and multidirectional reflexive processes based on real practical contexts stands out amongst the benefits of USL that have been found (Bringle and Hatcher, 1995), as do the development of personal, social, and civic competences (Bringle and Clayton, 2021; Celio et al., 2011; Salam et al., 2019) and the fostering of social justice (Moely et al., 2002). Ultimately, USL favours the comprehensive training of university students because it combines theoretical and practical learning by university students in more real contexts, leading to improvements in their professional competence (Chiva-Bartoll et al., 2020; Maravé-Vivas et al., 2022). These aspects are decisive for adopting an inclusive focus as future professionals.

It is very important that people who are users of USL projects also benefit from them. In this sense, there is considerable research that advocates for the horizontality of all of the stakeholders participating in SL projects, including the people and bodies that use them (Monjas et al., 2022). Research has also been found that encourages collecting information from the users of the service in research, as what these people might report is unlikely to be reported by the other participants in the SL projects (Calle-Molina et al., 2022; López-de-Arana-Prado et al., 2023). In most cases, these people live in vulnerable or marginalised situations and so there is a need to offer them a space where they can express their viewpoints and their opinions to the other participants in the project and to the research collective (Calle Molina et al., 2024). In fact, the people who research and design USL projects should reflect on the need to select the most appropriate tools for effectively gathering the perceptions and viewpoints of the people who receive the service. Consequently, the choice of these instruments must be adapted to the particular characteristics of the people who receive the service, for example, people with intellectual disabilities or people who experience difficulties communicating their opinion of the project. If care is not taken with these aspects, the functioning of the SL project and its benefits could be compromised. Curiously, the specialist bibliography contains very few studies on USL that reflect the impact of this methodology on people who use these services, as most of these studies centre on identifying the impact of this methodology on the professional, social and personal skills of the university students who participate in this type of project (Pérez-Ordás et al., 2021).

Using acrosport as content requires cooperation and interaction between participants, which involves the development of social and affective skills (Abós et al., 2016; Ávalos et al., 2020; de la Osa and Gutiérrez-Sánchez, 2015) whilst at the same time requiring participants to face challenges and solve problems, taking the personal characteristics of all of the participants into account and demanding self-improvement and the self-confidence to face and overcome the challenges posed. Resolving challenges collectively requires negotiated and democratic decision-making.

The aim of this research is to identify the effects of an inclusive acrosport programme delivered by students from the bachelor’s in physical activity and sports sciences for university students with and without intellectual disabilities using the service learning methodology.

## 2 Materials and methods

### 2.1 Participants

Students from three education programmes at the Autonomous University of Madrid between the ages of 18 and 23 participated in this project: five students (two women and three men) from the second year of the university bachelor’s degree in physical activity and sports sciences (PASS); five female students from the fourth year of the bachelor’s degree in primary education with a specialisation in physical education (BPE-PE); and 15 students (eight women and seven men) with intellectual disabilities from the second year of the Training Programme for Labour Inclusion (TLMIC), also called the “Promentor Programme,” a university programme aimed at young people with mild intellectual disabilities for their future labour market inclusion.

Regarding the roles played by the participating students, the PASS students received acrosport training in the subject “Fundamentals and Didactics of Individual Sports” and subsequently designed and implemented an inclusive acrobatic training programme. The PEF-PE and TLMIC students who received the inclusive programme had no prior experience in acrosport.

Concerning the selection criteria, all participants in this study had to be enrolled in one of the degrees or subjects mentioned in the previous paragraphs. PASS and PEF-PE students also had to be willing to participate voluntarily in the project. All students enrolled in TLMIC were selected.

### 2.2 Methodology and instruments

The research used a qualitative methodological design, adopting a case study approach that made it possible to identify a rich and detailed perspective of participants.

Following Lincoln and Guba (1985), the criteria of methodological rigour that this research has followed include credibility (textual citation of transcripts and triangulation of data, researchers, and methods), transferability (description of the context, participants, processes, and conditions of the study), dependence (detailed record of the methodological process, definition of categories, and application of systematic coding using NVivo Qualitative Data Analysis Software to organise and track the coding process), and confirmability (presentation of evidence with textual citations, triangulation between coders, and coding agreement, carried out by two researchers who coded the same interviews independently and subsequently discussed their interpretations).

Four instruments were used to collect information in this research:

1. Structured interviews (I), allowing in-depth exploration of the areas of interest for the study, were carried out by the PASS students who designed and implemented the SL project.
2. A focus group (FG) in which the BPE-PE students who attended the acrosport classes participated.
3. Field journals (FJ) completed by the PASS students who designed and implemented the SL project.
4. Learning journals (LJ) filled in by the students with intellectual disabilities who attended the acrosport classes. The learning journal was cognitively accessible. This feature allowed for students’ independent work and ensured their engagement.

### 2.3 Analysis

The focus group and the five individual interviews were recorded so that they could be played back and transcribed, and their content analysed. This information, along with the information obtained from the field journals and the learning journals, was analysed using the NVivo Qualitative Data Analysis Software (version 15.1.2.18).

Descriptive and interpretative analysis was performed. The following process was used to conduct the content analysis of the qualitative data: Relevant segments from the data that represented important or recurring ideas were identified and coded. The codes were then grouped into broader themes with shared ideas from which the central categories and subcategories emerged.

Before starting the study, permission was requested from the director of the Training for Labour Market Inclusion course to involve the participants. Written informed consent was obtained from people who participated in the study for the publication of any potentially identifiable images or data included in this article. The purpose and scope of the research were clearly explained, including the procedures to be followed, the voluntary nature of participation, and the measures in place to ensure confidentiality and data protection. Furthermore, the director reviewed the study protocol and granted approval, allowing the research to proceed in accordance with institutional guidelines. Undergraduate students were informed about the study and provided their informed consent to participate via a document signed, in line with ethical research standards.

## 3 Results

Table 1 shows the list of categories, definitions, and subcategories resulting from the analysis, as well as the number of references to each of them.

**Table 1:** Categories, definition, and subcategories.

Categories, subcategories, and definitions	References
Initial motivation (Motivations of PASS and BRE-PE students to participate voluntarily in this project)	11
Prior experience (Previous experience of PASS and BRE-PE students with ApS and/or intellectual disabilities)	25
Service learning	7
Intellectual disabilities	18
Learning acquired (Lessons learned during the project in relation to acrosport or other areas)	98
Acrosport	62
Other learnings	36
Design and implementation (Process of planning and implementing acrosport sessions to achieve the proposed objectives for the students)	49
Adaptations (Modifications to facilitate access to learning according to individual or group needs)	37
Transference (Applicability of acquired learning to academic or professional contexts)	0
Academic	13
Professional	17
Inclusion (Benefits, barriers, and challenges encountered in the process of achieving that all students felt valued and could actively participate in the learning process)	60
Benefits	40
Barriers	8
Challenges	12
Stereotypes (PASS and BRE-PE students’ prior perception of people with disabilities and their development)	14
Relations (Connections created between different groups of students)	59
Student-to-student	21
Teacher-to-student	38
Expectations (Previous expectations of PASS students regarding participation in an inclusive ApS project)	14
Feelings (Sentiments experienced by all participants during and at the conclusion of the project)	63
Recommendations (PASS and BRE-PE students’ consideration of the project’s usefulness and recommendations to other students)	16

### 3.1 Initial motivation

There were differences in initial motivations for participating in the project between the PASS students in their role as teachers and the primary education students in their role as students. The former group reported that their motivations for choosing to participate in the project related to previous positive experiences of participation in service learning or experience with people with disabilities that they wanted to repeat.

it was a way of starting to teach and so you were introducing yourself to the world of being a teacher, so that was very good because we had to do a piece of work as teachers and we had to see on the fly if we could adapt the exercises and everything and I really liked it last year so I said… that this year I also wanted to do it (MJG_I).

last year we only had one session, but I think that I learnt quite a lot and it was very useful (MFG_I).

In other cases, they noted that other fellow students had participated in SL projects and recommended the experience.

Last year my classmates did SL and that told me that it was very good, that they really liked it (JGM_I).

In contrast, the primary education students, in their role as students, felt other types of motivation. In some cases, they perceived shortcomings in their initial training relating to disability, and they saw a training opportunity in this experience.

For me mainly because the module that we did maybe about students with needs came during COVID, so I, at least, didn’t make as much of it as perhaps we should have (LS_FG).

the educational psychology for the inclusion of students module, fell a bit short for us. And that …When meeting each other and seeing how we are really going to work with the students, you could see that the practical side of the module was very limited (ML_FG).

Another motivation was to meet a group they had never mixed with and which they do not know, or because they feel that they have some prior stereotypes about people with disabilities, and this experience could help break these down.

personally I did not mix much with people with needs so, in this case I also like to know how they relate to one another, what their concerns are. In the end, they are the same age as us, so they have more or less the same concerns as us (SF_FG).

I think that attending this type of classes you also see a bit of their abilities, and we sometimes have stereotypes that they will not be able to do “x” thing (LS_FG)

Then when you see it, as LS says, the thing is that all of your stereotypes fall away and so you can put yourself more in the role of students and teachers when it comes to relating with classmates and how you learn (ML_FG).

In this sense, they also believe that there are personal aspects that they could improve by participating in this project, which serves as motivation to participate voluntarily.

I also like to know which ones they are and how they are developed amongst them, above all socially, because it seems to me that their level of empathy is incredible, it is very high in some cases. And that seems very important to me, because I often lack empathy. So it seems very important to me to see how they focus it and ultimately they are even in many cases better people (SF_FG).

### 3.2 Prior experience

Regarding the prior experience of participants from the PASS and primary education degrees, two types were identified: previous participation in SL projects and previous contact with people with disabilities. In the first case, three of the students from the PASS bachelor’s had previously had the opportunity to participate in other SL projects.

last year I liked it a lot and because it follows the line of last year and because I think that I learn more from SL than what I can contribute to them (MRD_I).

However, two of them did not have previous experience. One voluntarily chose not to participate in the past but, on this occasion, decided to participate on the recommendation of his classmates, as noted in the previous section. The other did not take the module in which the SL project was offered, as it had been convalidated with previous studies.

as I had convalidated modules, so in the modules where it was done, I think it was done in hockey, I had convalidated it and I couldn’t do SL (ARP_I).

None of the students from the primary education course had previous experience relating to SL projects. Regarding prior relations with people with disabilities, the PASS students who had participated in previous SL projects had also done this with people with intellectual disabilities, albeit for a very limited time, and they also noted that they had not had experiences before this occasion. In one case, an experience in the field of sport was mentioned, and in others, experience in school placement.

in my school, which was the Pío Baroja, there was a programme which was the gardening programme, and on that it was above all people with intellectual disabilities. So then we had, I do not know if it was three sessions that we had to propose a session that was the first contact with them […] in general I do not happen to meet people with disabilities (ARP_I).

The primary education students had also had experiences with people with disabilities in placement schools, and they observed that there had been shortcomings.

when I came to the early childhood placement, I had students who had needs and at first how to engage with them surprised me a lot because I didn’t know how to do it or anything (LS_FG).

In another case, the previous experience was in an informal educational context, and so the student felt that there were significant differences with the context experienced in this project:

Me, for example, I have worked with children in my work in camps, but it is very different. When I worked with them, I didn't know how to act in many ways and when I see a session now you say, okay, there are lots of things you see, that you take into account, that you can apply…. (SL_FG).

### 3.3 Learning acquired

Given that acrosport was the content from which the sessions were conducted, many types of learning are reported. In the teaching role, the PASS students considered that the sessions were a refresher of what they had seen in the subject and they could consolidate this knowledge by developing the session in a real situation, with the learning being more meaningful.

I think that revising the topics we covered in class helped to consolidate concepts and explaining it to other people helps us as a study method, focussing on more specific things such as common mistakes (JGM_FJ_2).

above all, the help that we can offer with regards to the different blocks that perhaps when it us who do them, we do not come to understand why this help is necessary or learning these ways of helping, and when you are in a class and you put yourself in the role of teacher then you do understand why it is very important to underline how you have to help one another and what types of help are necessary for each thing (MJG_I).

In their role as teachers, they also affirmed that the students understood the acrosport content (safe execution, helping, holds, roles in forming pyramids, identifying errors, support areas…), reflecting on their own teaching practise to facilitate learning in the future for other students who did acrosport.

They have learnt the different holds and the positions, and even in doing the postures, they knew how to correct the bases (ARP_FJ_2).

From my point of view, the session went quite well as the students had fun and understood the main questions of helping and safety in acrosport (MJG_FJ_3).

As for the ways of making a pyramid, which are the areas where they have to do the supports, what I said before, being a top, being a base, what it means (JGM_I).

Moreover, the students without disabilities who received the service also mentioned their learning, not just of acrosport content but also what they learnt as future professionals to be able to plan an acrosport session, taking into account questions that they had not previously considered.

it gives you much more technical language that I did not know. It is true that with L. we did Acrosport, but not at this level in the sense of safety they gave us so much advice or at the technical level of how to do certain postures and also at the level of words, as in this sense I personally learned quite a lot (SF_FG).

And also at the level of doing progression of sessions in the school (ML_FG).

for example, when M. was there or maybe a team was put together, it was like they were also separated by their height, weight and so on. And so it was also like they gave you things on how to do a session. And that is an added value as professional (IP_FG).

Similarly, the students with intellectual disabilities who received the service explained in their learning journals what they learned about acrosport, specifically aspects such as types of holds, positions, supporting weight, the names of roles in acrosport, questions of safety, etc.

one was the base, the other is the top and the other two supervise the base and the top to make sure they do it well, these are also called helpers. Before doing the poses, you have to be clear about certain things, we always get onto the base’s hip, but we never get onto the other person’s back because we could injure it (EP_LJ_3).

Then we did before the shape of the pyramid, we did the hold in lots of ways to support the body weight claw hold, supporting one another (MA_LJ_2).

we have done lots of work in a team for example making a pyramid one on top of the other you had to have lots of communication and we also help one another to put ourselves in different postures and do it well to avoid injuries and hurting other classmates and supporting the body weight (MA_LJ_3).

I have learnt new words like base (which is the person who supports the weight of the other people) and top (who are the people who climb on top of the bases) (PJ_LJ_3).

### 3.4 Design and implementation

The students who took on the role of teachers identified an increase in their knowledge of how to design and implement a session, competences that they have to acquire as future professionals in physical activity and sport. They reflected on adaptations taking into account the observations that they listed in the field journal, the difficulty of the tasks, handling timing, groups, organising the material, feedback, individual support, coordination as teachers in the classroom, questions of autonomy, etc.

We had thought about a series of exercises that we changed as they were doing them very well and very fluently. for example, the exercise of holding the weight of the classmates, we had thought that they would not go below 5 people holding one, but seeing how they were doing it, we thought that they could even be put in smaller groups, down to 4 people (ARP_FJ_1).

activities that we thought that they would not be able to do because of lack of time, we moved on to the last task and in the end we had to adapt it because they went too quickly and there was going to be too much time (ARP_FJ_3).

As for the organisation of material I think that it went quite well, but it is true that as for time it is possible that we dedicated more time to some phases than to others owing to how much we could develop each activity (JGM_FJ_1).

I think that we have to be very much aware of the training of groups so that they can help people who have more difficulty with strength or balance, I think that this first session was very useful to us to see what the students were like and in preparation for the next session be able to make certain exercises easier or harder (JGM_FJ_1).

This session was very useful for getting to know the group; what groupings are better, who is more outgoing and shy, who is more active in the class, etc. It was a necessary first contact with them to be able to improve and adapt to them in the following ones (MFG_FJ_1).

Our organisation was quite good, as we tried to organise the material between all of us and provide feedback to the students in each activity (MFG_FJ_1).

In the next session we could incorporate games that reinforce the concepts learnt. Encouraging creativity and active participation (MRD_FJ_2).

I was attentive to the groups to make sure they didn’t hurt themselves doing acrosport as safety is fundamental. I also tried to make sure it was not just a correction, but that they understood what they were doing wrong so that in future they can correct it themselves (MJG_FJ_3)

basically the organisation of the groups, which people had motor disabilities so that they were not put together, and then also what people had a problem with other people or who could not be together (MJG_I).

Regarding the quotes collected, it is apparent that their reflections after the third session comprise a more positive self-perception of their role as teachers compared to the previous sessions:

In this class, I kept a closer eye on one group, which V. was in, who was the student who had the most difficulties. I helped her to position herself and correct her body position and include her in the session. Also, to be sure that the rest of the members of the group were also doing it well and safely (MFG_FJ_3).

The organisation was better than in the previous sessions, as well as the dynamic used. It was easier to be attentive to each of the groups as one of us could be with each group (MJG_FJ_3).

### 3.5 Adaptations

The students, in their role as teachers, identified the adaptations they made to the acrosport sessions as something very important, as the design of the sessions and their implementation had to be adapted to the characteristics of the participants. In general, the adaptations they made relate to visual resources and simpler explanations, a simpler series of activities in case the ones they proposed turned out to be difficult, explanations and communication, the groups taking into account the level and the intergroup relations, repetition and constant feedback, and specific adaptations for various students with motor or attitudinal difficulties. In some cases, the adaptations are mentioned as something they did not take into account because of their lack of experience, but that served as a learning experience and source of reflection for them.

we did not realise, M. told us, in the first exercise with the cones, as V had to bend down, because she couldn’t reach so for the next time, for V. or whoever, we will adapt the tasks so that they do not have any difficulties (ARP_FJ_2).

I would like to know how to deal with situations like the one with J. Because it is true that we were encouraging him and at first she started well, but there was a moment when we had to ask M. for help to be with him (ARP_FJ_2).

In other cases, they mention the capacity for adaptation as something that they took into account during the sessions in a forward-looking way, in case it was needed.

the first session that we had, as we did not really know what each person's abilities were, we planned a slightly more general session, but then, as we have moved through the sessions, we have seen which people have more difficulties, where they have difficulties and we have designed activities, variants for these people (ARP_I).

maybe they found it hard to understand one of the exercises because of the words we used, which perhaps were difficult, even for us they were hard, as we tried to explain it to them in a different way, we tried to explain it with drawings, a simpler explanation (ARP_I).

creating balanced groups as there are people with motor difficulties or who find communicating harder and this latter thing is very important. Also, so that some students don’t get distracted or so that they remember what the most important thing in the session is, repeating it several times (MJG_FJ_1).

Some of the people with reduced mobility found it hard to get into this position and so we provided an observer so that they can participate in the session as well as changing the position of part of the body to make it easier for them to do it (MJG_FJ_2).

we used the projector because … their being able to see it helped us so that we hardly had to make practically any variations or no variation. The variation came afterwards during the exercise, but in the explanation, having the projector, it is adapted to everyone (MRD_I).

It is either posed as something that they had changed or that they would like to change on future occasions.

For the next time, what we have thought about doing is making a series of pyramids for the ones who are most advanced and for those who have more difficulties, some simpler ones, but that all of them can do pyramids, even if they are simpler (ARP_FJ_2).

In general, they note that whilst the session was designed and adapted for all of the students (primary education students and students from TLMIC), one primary education student was included in each group so that, as well as students from the two programmes being able to get to know each other, they would be a helpful resource to be able to do the adaptations that the PASS students had designed on some occasions:

I think that we simplified everything as much as possible so that it was as easy as possible to understand for everyone, both for Promentor students and for the primary education students. So, the one thing that we perhaps might have sometimes been able to use is that there would always be a person from primary education in each group to try to make sure that if there was a problem, a need or whatever, this person might also be there as a helper in some form (JGM_I).

### 3.6 Transference

The participants in this project consider that there was transference of their learning to their future employment. In the case of the students who acted as teachers, they also report that there was transference of their learning in the degree to the SL project. They consider that this is more of a training experience than a theoretical exercise as they learn from having to manage situations in real practise.

For me it was a great experience, I think much more educational than having done a simple project, putting into practise the proposed sessions I think helps us with our first, development educationally and professionally (JGM_FJ_3).

They note that they feel their learning is greater than that of the other students from their course who decided not to participate in the SL project, as they have revised the content in practical situations.

in the end what we do we learn and we put it into practise and them no. Well I want to be a teacher and so that aspect will also be good for me for (MFG_I).

They consider that the learning acquired in this project transcends the acrosport content, as they have experienced situations that might occur in their future teaching work.

in the end as future teachers some of us then will have cases where there are people with disabilities in class and so in the end with these activities well you learn a bit about how to organise yourself how to make sure the person is included in class (MFG_I).

you have to explain and if they don’t understand you have to find another way to explain it, so in the end you are absorbing the concepts better … because if you have to explain it in different ways so that they can understand in the end you understand it better (MJG_I).

On the other hand, the students without disabilities who received the service, in their role as students in the SL project, consider that with this practical experience, they have been able to transfer and understand concepts like “inclusion” that they had seen in theory in modules.

I personally think that we are often told about inclusion, well, not often, very often we are told about inclusion and nobody puts us in a context and nobody has made us be inclusive at any time and being participants in what is a truly inclusive session. When I went to the class, because maybe before I didn’t think so, but now I have been I think this should be obligatory. So, we have this opportunity in the university and from the class five of us went, six of us, from the 120 who enrolled for year five of the degree (SL_FG).

### 3.7 Inclusion

The fact that the sessions were inclusive and aimed at students with and without disabilities was something the participants identified as important in this project, noting the benefits, barriers, and challenges generated.

With regard to benefits, the students in their role as teachers, despite having planned inclusive sessions, were spontaneously supported by the primary education students, as the groups were designed so that there was always a primary student in each group to favour inclusion. In this sense, they consider their help to have been positive, even though it sometimes did not contribute to the sessions being inclusive, as their role should have been that of students.

them too, as they are from primary education, they played the role of teacher a bit, because although they were also supposed to be students, that is to say, they had to participate in the session … often, in the end they took on the role of teachers when they were in small groups to help them, so perhaps for this part it was a bit beneficial, because it will be what they want to do in future. But as for being a student in an inclusive class, I think not so much (MJG_I).

One of the benefits that they underline from the inclusive sessions is the normalisation of diverse abilities and disabilities, regardless of the role or group to which someone belongs.

the fact of working with people with disabilities and the students from teaching, I think that the act of normalising the situation of the SL group was fostered, even though they have the disabilities they have, it can work as one person, like other people, and they don’t have any difficulties. It is true that maybe you have to help a bit, but they are independent (ARP_I).

In this sense, the students who delivered the sessions as teachers consider that inclusion was beneficial for the students without disabilities with regard to communicating with students with intellectual disabilities, as they will be part of their life as teachers in the future. At the same time, they mention the fact that there are new people in their classes as a motivational factor for the students with intellectual disabilities.

knowing how to communicate and so on and in the end I also feel that for them it can be a… as they are going to be teachers, then, a good tool having this contact with people with disabilities (MFG_I).

I think that for the Promentor students, they really enjoyed themselves, I think, or they really enjoyed the primary education students being there. I think that also it was like a motivational factor, they really wanted to go with them too, they did not just stick within their group (JGM_I).

At the same time, they consider that the benefits derived from inclusion in the sessions meant that their learning, in addition to the acrosport content, was very rich in other fields such as empathy, the capacity for effort, the good classroom climate, and ultimately social and emotional aspects that, if they had not been inclusive sessions, would have been reduced.

And their capacity for effort really caught my attention […] We often complain or say that we can’t and there are people who have to make much more of an effort (LS_FG).

And then I felt really welcome, because it is true that, perhaps with us, when maybe someone comes from outside because they still have to finish a module or something, it's like he or she is the person who comes on their own to this class, but like they don’t become included because we don’t help them to include themselves. And us from the first day I felt super welcome. All of them came to ask what you were called, what you studied, or perhaps they would tell you their name (LS_FG).

I think that the content as such, with what SF said, you would have learned it the same, because they did not influence whether you learned more or less, but the more emotional or more social part would have been much more reduced (LS_FG).

To finish, they observe that one of the benefits of the inclusive sessions is the creation of connections within the university community, an aspect that lasts over time, even though the project has ended.

you’d go through the faculty and you don’t realise and you look and you say “oh, here are the people from the project who ate next to us in the cafeteria and there are the people from PASS,” you hadn’t realised. And so it is kind of like now you also notice more and you are aware that there are other types of people who not only are in your primary teaching class, and it is kind of like you put a face to them and you are aware of them (SF_FG).

when we see them in the corridors, they recognise us, we recognise them, we say hello (LS_FG).

Although most of the participants appreciated the benefits of the inclusive sessions, some barriers were mentioned.

I think that both have benefited, because in the end they acted a bit as teachers and helped them to do the exercises, but it was also a hindrance because they got distracted more. So as lots of them were more distracted, they didn’t listen to lots of things that we were explaining … […] there were times that the students from primary education spoke more or got more distracted than the actual students we were giving the session to (MGJ_I).

Regarding the challenges raised by the sessions being inclusive, the students who acted as teachers identified some specific questions of support for particular students with intellectual disabilities to which they did not feel prepared to answer.

There was one student who I often did not know how to act with, as he was not well emotionally to do the session (MFG_FJ_2).

I tried to include him in the group, but I could not and did not really know where to start… (MFG_I)

the situation worried me a bit, what might happen, whether she could continue to do the session, because also she was enjoying it, she was a girl who was really animated, that she wanted to carry on doing the session, but that bothered me, like an internal frustration that she couldn’t continue with the session (ARP_I).

On the other hand, the students without disabilities, in their role as students in the SL project, consider that from this practical experience they have been able to understand questions like “inclusion” that they had seen in theory in their modules.

we are often told about inclusion, well, not often, very often we are told about inclusion and nobody puts us in a context and nobody has made us be inclusive at any time and being participants in what is a truly inclusive session. When I went to the class, because maybe before I didn’t think so, but now I have been I think this should be obligatory. That is to say, we have this possibility within the university and from the class five of us go, six of us, from the 120 who enrolled for year five of the degree (ML_FG).

Finally, the students with intellectual disabilities felt motivated when doing physical activity with people whom they did not know, and they felt it was very interesting and fun.

Today I felt very motivated as we did these activities with other education students who we did not know (EP_LJ_1).

this type of exercise was very interesting and fun, whilst it explains gymnastic skills to the education students (JA_LJ_3).

### 3.8 Stereotypes

The students without disabilities who delivered and received the service considered that they had prior ideas about students with intellectual disabilities that, in practise, were gradually overcome. For example, they considered that the disabled students’ intellectual or physical capacity was less, and some of the non-disabled students noted a degree of concern about being unable to cover the content because they thought that the disabled students would not be able to do it.

sports I think that in the end people very much see it as a taboo that people with intellectual disabilities, for example from a motor aspect, can’t do the same as us but really they can. Because in the end they have an intellectual disability and you have to explain it to them differently but that’s it, they can do everything else (MJG_I).

attending the classes I realised that it is practically a “normal” class, that they can do all sorts of positions, in this case that were Acrosport (LS_FG)

This is what LS was saying before about completely getting rid of our prejudices. For example, people often say an autistic person can have lots of difficulties when relating socially and we did not have any type of difficulty. The problem is the way you have to approach them and build this relationship, I think (ML_FG).

In some cases, they even noted that the students with intellectual disabilities have better abilities to communicate or relate than they do.

I thought that their ability to relate to people would be much lower than they have. I thought that they would find it much more difficult at the level of speaking and at the level of expressing themselves controlling their emotions a lot. They know how to recognise them really well, in fact, I think that they recognise them a thousand times better than me and they relate a thousand times better than me at an emotional level and this impacted me a lot and above all the empathy they have […] personally I think that they are well practised in all of the senses, at an emotional level, in communication and this surprises me a lot (SF_FG).

One of the particular features that they noted is the students’ engagement in the sessions, which differed from the general attitude of their classmates from the PASS degree that they are studying.

The session helped me see that the students’ predisposition for learning the acrosport content. You could see lots of motivation on their part when doing the different positions which is not very common in other classes of students (MJG_FJ_3).

The students who provided the service thought beforehand that the sessions would be very different from those they received in their degree programme; however, afterwards, they reflected more on the similarities than the differences.

and often they are more motivated than us to do it, and people say “oh no, they have intellectual disabilities so maybe they can’t do anything and I don’t know how I’m going to have to design the session” but in reality we designed sessions that were very similar to what we did in class. And they did them perfectly (MJG_I).

### 3.9 Relations

The students consider that the atmosphere in the sessions and the relationships between the participating groups of students were very good. They believe that a high level of trust was created, above all between the students with and without disabilities who received the service.

Amongst all of us we have created an environment of trust, in which the students felt comfortable expressing themselves and experimenting […] Relations of trust were established between the Promentor students, the ones invited from primary education and the teachers. The students showed respect and collaboration between them, amongst all of us we have managed to create an atmosphere of trust and support (MRD_FJ_1).

In this sense, the students who delivered the service saw differences with other SL projects in which they participated, as they felt that the students with intellectual disabilities built more trust with the primary education students, as they were sharing the role of students, than with those who played the role of teachers.

Perhaps a bit different this time because this time the SL was inclusive, the previous ones were not inclusive and of course, between them they have formed more of a group than with the teachers […] With regards to the previous time, in this one there was more of a group amongst them owing to the relationship or how the students associate with each other (MRD_I).

In the role of teacher, the PASS students consider it notable that the group relations between the students with disabilities were positive.

I think it surprised me that there was so much support and kind words between different people. I have not seen any arguments […] in the sessions everything was always very collaborative, if somebody hurt themselves, all of their classmates went to support them to see how they were, and nothing, everything was fine, I have not seen any long faces (JGM_I).

In this sense, they consider that it is the students with disabilities who enable personal relations to be simpler in class.

I think it was much easier including with the Promentor students, it was like very easy to relate to them […] I think that with them there was no need to follow a strategy or anything, simply them just as they are, as they came to speak to us and so on, we spoke to them before class, after class too (JGM_I).

Regarding relations, it is also noted that there is a relation that transcends the link that exists in the classroom, although they consider that they do not manage to establish a relationship outside the classroom owing to the limited contact time they had in the project.

well I think that in the end, we only know each other a little bit, but now whenever I see them I say hello and they say hello to me and I understand that they do it with my coursemates and the students from primary education too so in the end they do know more people in the university and that is always good and I don’t know how they open up to other people (MFG_I).

The students without disabilities who received the service have had more time relating to the TLMIC students, and they note some questions that posed challenges for being able to relate in the way they considered appropriate: trying not to infantilise their classmates from TLMIC and setting boundaries in uncomfortable situations.

I think that we often tend to infantilise people with disabilities and the thing is I don’t feel that I have done it, but it is true that maybe they approach you in a way that might be different to how we do it, because it is a reality and then you respond in accordance with this way that people are relating to each other (ML_FG)

In the end, it is something that I don’t like. And I also have to tell you that I don’t like this. I think that they also have to understand what people don’t like (IP_FG).

Finally, the students with disabilities who received the service observed that they thought it was interesting that the activities helped the students to get to know one another better, that they enjoyed themselves with their classmates, and that they found working in a group interesting.

Today we learned in more detail ways of getting to know each other in the gym session with team activities so that we get to know each other better (EP_LJ_1).

I liked it lots and above all it was very interesting learning how to work in a group (JA_LJ_1).

### 3.10 Expectations

With regards to the prior expectations of participating in an SL project, there are various opinions. Some of the students who provided the service initially believed that it would be more complex than it was because the students with disabilities facilitated the teaching practise through their high level of attention and motivation, and so they felt that the teaching practise was easier than they expected.

they have a good predisposition, they are in a good mood, they take everything well, they are eager to do the things. So for me it was very easy, very simple (JGM_I).

we imaged it would be much harder than it turned out to be. And we were very wary of doing these things, but I think that then when you have the experience you see that it is not so complicated (MJG_I).

In other cases, in their role as teachers, they underestimated the capacities of the students with intellectual disabilities.

as the sessions have progressed we have seen that they were achieving the objectives…, aspects perhaps a little more complicated like the helpers and where they had to position themselves on top people they also knew how to complete them […] I thought that they would find it harder, but after working with them, it even surprised me (ARP_I).

Some of them also said that their expectations had been fulfilled and others that they were surprised because they were exceeded, as the students with intellectual disabilities easily achieved the objectives:

More than anything because it was content we had seen in my own class and we went much more slowly than they did. So we thought that in these three sessions reaching this end goal (which was to build pyramids in three hours), was a difficult objective. And in the end we saw that in the last session we even had to add exercises, variants, because we had planned a session that was maybe a bit easier than what we though they were going to achieve. And in forty minutes they completed the session and carried on doing variants and surprised us (ARP_I).

### 3.11 Feelings

In general, the feelings of the students who provided the service evolved as the sessions progressed. At first, they felt nerves, tension, or insecurity; however, as they had more contact, they experienced feelings such as calmness, comfort, or enjoyment.

Yes, I think that it has always gone a lot from less to more, including, obviously, the first session we were a bit more tense, a little bit more nervous. In the end I did not know the group nor did I participate the previous year, so it I am a little bit more tense to see how it will be. But anyway, the truth is that from the first day they made it really easy because they brought willingness to do all of the activities, they embraced everything like “this is cool!” whilst we were thinking: “maybe they won’t like some activity, it is not the sort of activity they feel like doing” … (JGM_I).

Some of the reasons for which they experienced feelings that they identified as positive are that they considered the group of students to be very engaged in the sessions, very motivated, and making a lot of effort to do the tasks. This made them feel grateful in their role as teachers.

As in the previous ones, I felt very comfortable as they are a very nice group and it was very easy to work with them. Also, there is the case of V who despite having some aches and pains made a big effort to be able to do the session and this is something that is really appreciated. But in general, they are a very easy group to work with, as they constantly pay attention and are very eager (ARP_FJ_3).

Also to really thank the Promentor students who really got involved in the sessions and they were always motivated to do all of the activities that we had proposed (JGM_FJ_3).

Despite this, they felt frustration, worry or insecurity when there were circumstances in which they had to address the specific behavioural needs of a student, as these involved disruptive behaviour in class or a physical inability to perform a task, and they did not know how to act.

well it depends, when for example with J, I got a bit frustrated because I did not really know where to start and, so, at that moment, I didn’t know what to do […] frustration with the situation with J. and, then, emotion like I don’t know. Satisfaction (MFG_I).

In the simulated teacher role, they experienced sensations that were new for them. For example, the fact that the students thanked them for having prepared the session, the satisfaction of seeing that the students had understood the tasks and had fun in the session that they designed, or the importance of the students collaborating so that the session could go well. This last aspect was something they reflected on from their position as current students.

I think often when the session was ending and we were in a circle and they started thanking us for having given them a class, in the end that made me feel cheerful and happy […] I was fairly surprised because they not only worked, but they were more motivated than us to do the session. The truth is that you feel quite … in the end almost all happiness, but you feel quite cheerful because you often go to a class, you say that you are going to do acrosport and they do not want to do it and the motivation that they bring in the end helps you not only in the session, but you also realise lots of things… (MJG_I).

In the role of students, the participants from the primary teaching degree reported being very comfortable, although one of them, owing to her personality, initially found it hard to feel included in the group and socialise.

It is true that at first I did find it harder, I did not mix with them… and then it was like I always feel a bit scared socialising at first… like in a group that you do not speak for anyone and such like. But then I saw Sara [Promentor student] mixing so naturally that I said, right, this is a safe setting. Not because of prejudice, but also because of the way I am, with caution or insecurity or whatever… (ML_FG).

I did not have any problem, I was very happy with everyone, I socialised a lot with them, which in the end was what I wanted, to see how they related with other people and also with each another (SF_FG).

However, one student felt overwhelmed because a student with intellectual disabilities kept asking to be with her, and she felt that he was invading her personal space and did not let her connect with other people. She did not know how to manage this circumstance, and she expressed her feelings about this as follows:

at first fine, that is, all of the classes very good, but the thing is the last class, at the end, because of what I mentioned, I did feel a bit bothered by him […] but I do not know what to do so that he understands without hurting him, you know? And then, of course, I did not pay him attention, perhaps not attention, but I was also with other classmates and he took it badly. So I had a good taste in the mouth, but it is true that this did bother me a bit, saying perhaps you do not see it in the same way as me (IP_FG).

Finally, the students with intellectual disabilities expressed feelings such as motivation, tiredness, enjoyment, interest, calmness, relaxation, eagerness to learn new things and work in a team…

Today I felt a bit tired because they were not easy activities and I had to make an effort when I had to be a base (EP_LJ_2).

I liked the physical education class because I am learning new things and I want to go further in life. I liked the tatami and we want it to be repeated more […] I felt content in the university and learning is wanting (GS_LJ_2).

I had a great time and I like doing positions […] I like positions a lot and I do it at home with my sister (IF_LJ_2).

I felt good, relaxed, calm and the physical education class is very interesting (JA_LJ_2).

I loved the class and I had fun I felt very good (MH_LJ_2).

I felt very good but tired (MR_LJ_1).

I liked it a lot and I had a great time with my classmates (PJ_LJ_1).

### 3.12 Recommendations

The students who played the role of teachers would repeat the experience and would recommend that others do the same project. They consider that these projects are very useful for students who want to work in teaching.

yes, in fact, last year I recommended going to several of the people who participated this year and the truth is I would recommend doing it, as you learn a lot (MFG_I).

it should be much more important and form part of our academic curriculum or of one of the modules or something that is not just I do not know… but yes, I think that it should be part, you should have an experience, yes, because in the end lots of us will go and work in secondary schools will have that sort of student [with disabilities] (MRD_I).

Despite this, they note some difficulties with participating in this type of project as a result of a lack of academic time.

The problem we have is that as we are in the afternoon shift, if you have to work, because there are many classmates who work in the morning, so they have difficulties (ARP_I).

## 4 Discussion

Regarding the learning of content, it stands out that the students with intellectual disabilities who received the service said that they acquired a lot of knowledge, including learning to value working as a team (Kefallinou et al., 2020; Oh-Young and Filler, 2015). In addition to the above, the students who provided the service said that they learned more meaningful knowledge about the content than they did during the delivery of the module because they could apply it in real situations and because their participation in the project helped them reflect on their own teaching practise (García-Rico et al., 2023; Marttinen et al., 2020; Santiago et al., 2020). Finally, the students without disabilities who received the service said that they had developed learning relating to acrosport. These students also reported that this inclusive experience made them reflect on their own practise as future education professionals, including in their role as service receivers.

The students who delivered the service said that it increased their learning in relation to designing and implementing sessions (Lumish et al., 2022; Marttinen et al., 2020; Yorio and Ye, 2012). In particular, the students state that they feel more competent managing time in class, making groups, organising material, offering feedback and individual support, coordinating the teaching activity in the classroom, improving autonomy and making any adaptations required so that all of the students could achieve the expected learning. In relation to the adaptations, the students who delivered the service said that their participation in the project resulted in an improvement in their capacity to prepare, anticipate and adapt the sessions to the particular needs of the students with disabilities. These results agree with those of other previous research (Santiago et al., 2020).

In relation to the transference of learning, and in line with earlier studies (García-Rico et al., 2023), the students who delivered the sessions consider that their participation in the SL project consolidated their subject knowledge, and they underline that their learning is better than that of their coursemates who did not participate in the activity. These students indicated that all of the learning acquired during their participation in the project is more meaningful than what they learned in the theoretical classes and is highly transferable to their professional future. These results also agree with previous research (García-Rico et al., 2023; Santiago et al., 2020).

With regards to inclusion, the students with and without disabilities who received the acrosport sessions gave very positive evaluations of the class group comprising people with different capacities and from different university programmes. In particular, all of the students who received the sessions felt highly motivated by being able to meet people from other courses and by having the opportunity to provide mutual support and achieve the proposed objectives. The students state that these interpersonal relations were developed between peers, and the whole group saw them as normal, as the focus of attention was centred on doing the tasks (Moriña et al., 2020). The participating students also reported that the inclusive sessions produced benefits in the social and emotional environment, as the students stated that friendships in the class group developed that have continued over time and have developed outside the classroom. These results agree with previous studies (Berastegui et al., 2015; Klavina et al., 2014). However, challenges within the inclusive sessions were also identified, as the students who delivered the sessions recognised that they were not prepared to address some specific disruptive situations that occurred in the classroom and that it was not easy to redirect these situations so that the receptor students picked up the dynamic of the session again.

The students who delivered the sessions recognised that they had prejudices about people with disabilities, as they thought that people with intellectual disabilities would have numerous cognitive and social difficulties in doing the tasks. This underestimation of the students’ abilities meant that they designed sessions and activities that were too easy, and so when they found that the students with disabilities were more competent than they had initially expected, they had to redesign and adapt the tasks. These results are in line with studies that have shown that inclusive experiences reduce the prejudices of students who participate in this type of project (Lumish et al., 2022; Marttinen et al., 2020; Moriña et al., 2020; Park et al., 2024).

Regarding personal relations, the students who received the service maintained positive relations that inspired a high level of trust. The students without disabilities who received the service consider that the people with disabilities created a very pleasant atmosphere that encouraged establishing relations of friendship. Moreover, the students with disabilities reported that the experience taught them a lot and that they improved their capacity to relate socially. These results agree with those of other previous research (Berastegui et al., 2015; Bringle and Clayton, 2021; García-Rico et al., 2023; Lawson et al., 2017; Woodruff and Sinelnikov, 2015). On this same line, the students with disabilities said that they found working with students from other university qualifications interesting and enjoyable, as it enabled them to get to know other people.

Moreover, in line with earlier studies, the students who delivered the service reported that their feelings had evolved throughout the project. In particular, they stated that they felt nerves, tension and insecurity at the start of the project, but after time had passed, they said that these feelings changed into calmness, comfort and happiness. They noted the evolution of these feelings in the processes of reflection that took place throughout the project. The increase in their perceived self-efficacy as teachers stands out amongst the causes of the development of these feelings, as do the good disposition and motivation of the people who received the service. On the same line, feelings of happiness and enjoyment also emerged, caused by seeing how the students who received the service enjoyed doing the sessions that were designed and by receiving the thanks of the receiving students. These results agree with those found in other earlier research (Lynch et al., 2019; Marttinen et al., 2020; Pettigrew and Tropp, 2008; Santiago et al., 2020). Nonetheless, feelings of frustration, preoccupation or insecurity also emerged when encountering situations that demanded a higher level of experience and preparation.

The students without disabilities who received the service experienced feelings relating to comfort, thanks to the ease with which they could be included in the receptor group, but they also expressed feelings of being stressed by not knowing how to set boundaries in some specific circumstances with the students who have disabilities.

The students with disabilities who received the service expressed feelings such as motivation, tiredness, enjoyment, interest, calmness, relaxation, eagerness to learn new things, and work in a team. These results agree with previous studies that recommend reflecting the opinions and perceptions of the people with disabilities who participate in research (Calle-Molina et al., 2022; López-de-Arana-Prado et al., 2023).

## 5 Conclusion

The results of this research have demonstrated that the development of an inclusive acrosport programme using a service learning methodology has had positive effects on all university students involved. Specifically, the development of the project has fostered inclusion and social relationships free of stereotypes and prejudices, increased meaningful learning, and improved personal and professional skills.

The most important benefits generated by the development of this programme are presented in the following lines:

University students with intellectual disabilities acquired relevant knowledge about acrosport and improved their ability to work in teams, collaborate and socialise with other university students. University students without intellectual disabilities who received the service also acquired skills related to acrosport and developed their ability to identify areas they need to improve to become better teachers. University students who taught the programme reported that their sense of professional competence had increased significantly. Enrolling in the project allowed them to apply what they had learned in their degree classes, as they had to design, adapt, manage inclusive sessions and reflect on their own teaching practise.

Finally, the findings of this study support the implementation of inclusive programmes based on service learning as a comprehensive training strategy capable of promoting equity, social engagement, and the development of essential competencies for inclusive education and social transformation.

The design of inclusive service learning programmes allows students with and without intellectual disabilities to participate and interact naturally and on equal terms. These projects foster the creation of emotional and social bonds amongst diverse students that promote active participation, inclusion, and the elimination of prejudices and stereotypes.

Students who teach learn to plan and develop sessions with diverse students. Acquiring these professional skills is essential for developing inclusion, catering to diversity, and building more fair and respectful educational environments.

It facilitates the creation of emotional and social relationships amongst diverse students, promoting a climate of respect, collaboration, and mutual support for building a society more sensitive to diversity.

Service learning connects theory with practise, linking academic content with real-life situations that foster deeper, more transferable, and meaningful learning.

These types of initiatives represent an effective way to advance inclusive education, which not only recognises diversity but also values it as an opportunity for collective learning and social transformation.

The results of this research suggest that these types of projects are beneficial for inclusive education. However, it is important to note that the number of participants and the duration of the programme are limited. Therefore, the findings of this research should not be generalised and are not necessarily transferable to other educational contexts.

Due to these limitations, we propose increasing the study’s duration and the number of participants to know the evolution of the findings over time in a broader and more diverse population. Additionally, it would be interesting to understand the effects of developing similar projects implemented in other university programmes, other educational stages, or in other contexts. We also propose the use of mixed research methodologies that could enhance or complement the results achieved in this study.

## Data Availability

The original contributions presented in the study are included in the article/supplementary material, further inquiries can be directed to the corresponding author.
